# Supplementary figures and images for: From Feasting to Fasting: The Arginine Pathway as a Metabolic Switch in Nitrogen-Deprived Chlamydomonas reinhardtii
Source: Cells. 2023 May 13;12(10):1379. doi: 10.3390/cells12101379 (PMC10216424; doi:10.3390/cells12101379)

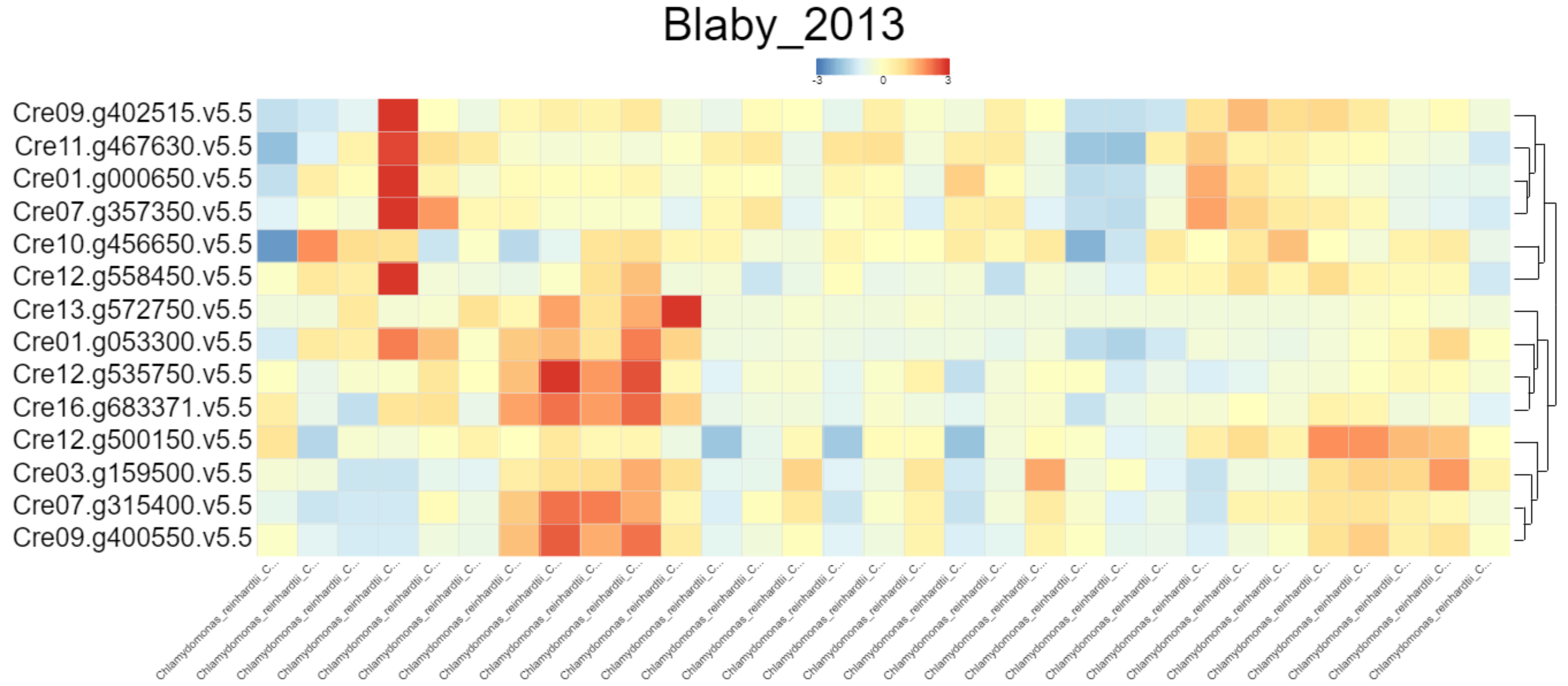

Supplement: Supplementary file 1 [file cells-12-01379-s001.zip › Figure S1, Blaby et al. (2013) -N transcriptomics dataset. Pathway = Arg catabolism to polyamines and NO.png]

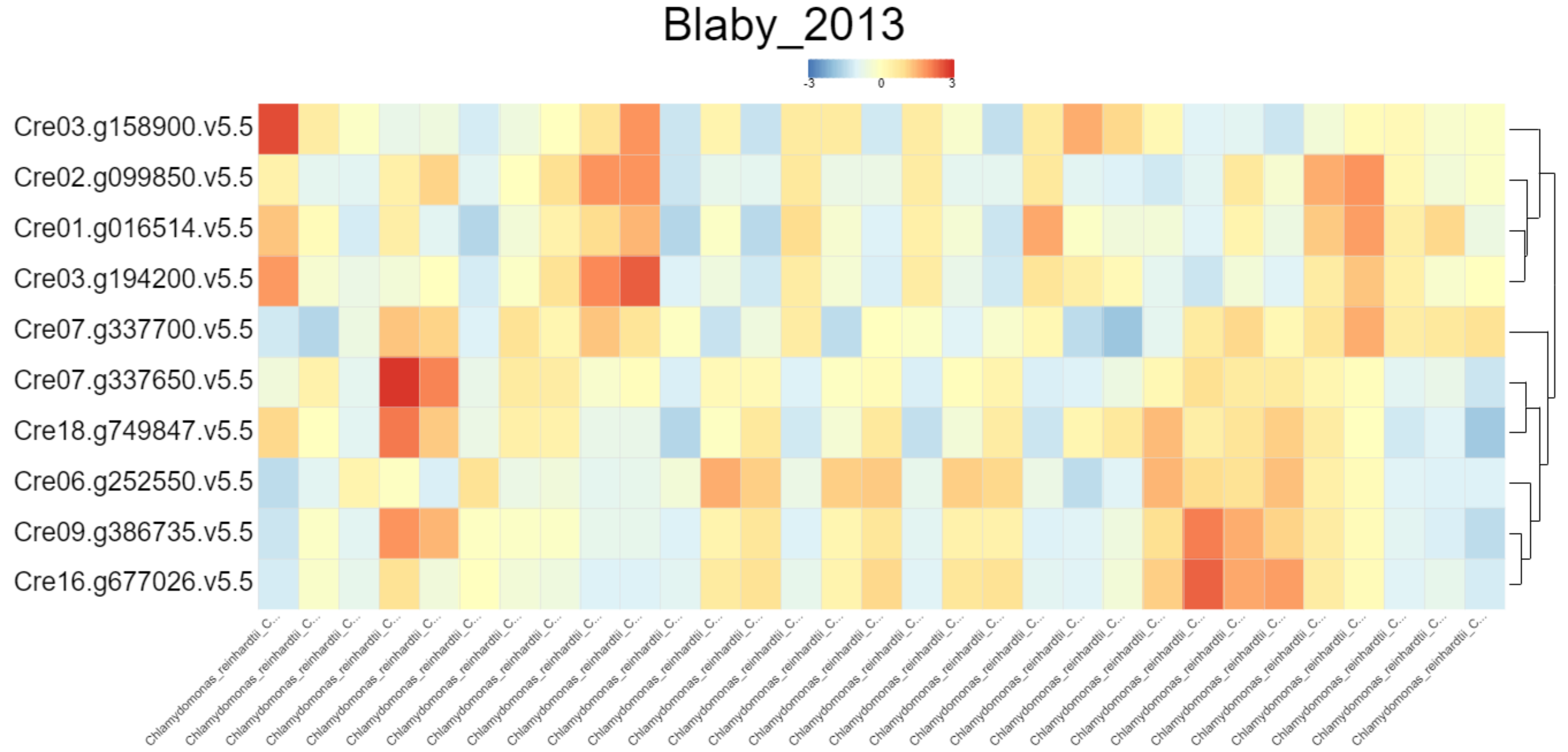

Supplement: Supplementary file 1 [file cells-12-01379-s001.zip › Figure S10, Blaby et al. (2013) -N transcriptomics dataset. Pathway = acetyl-CoA synthesis from pyruvate.png]

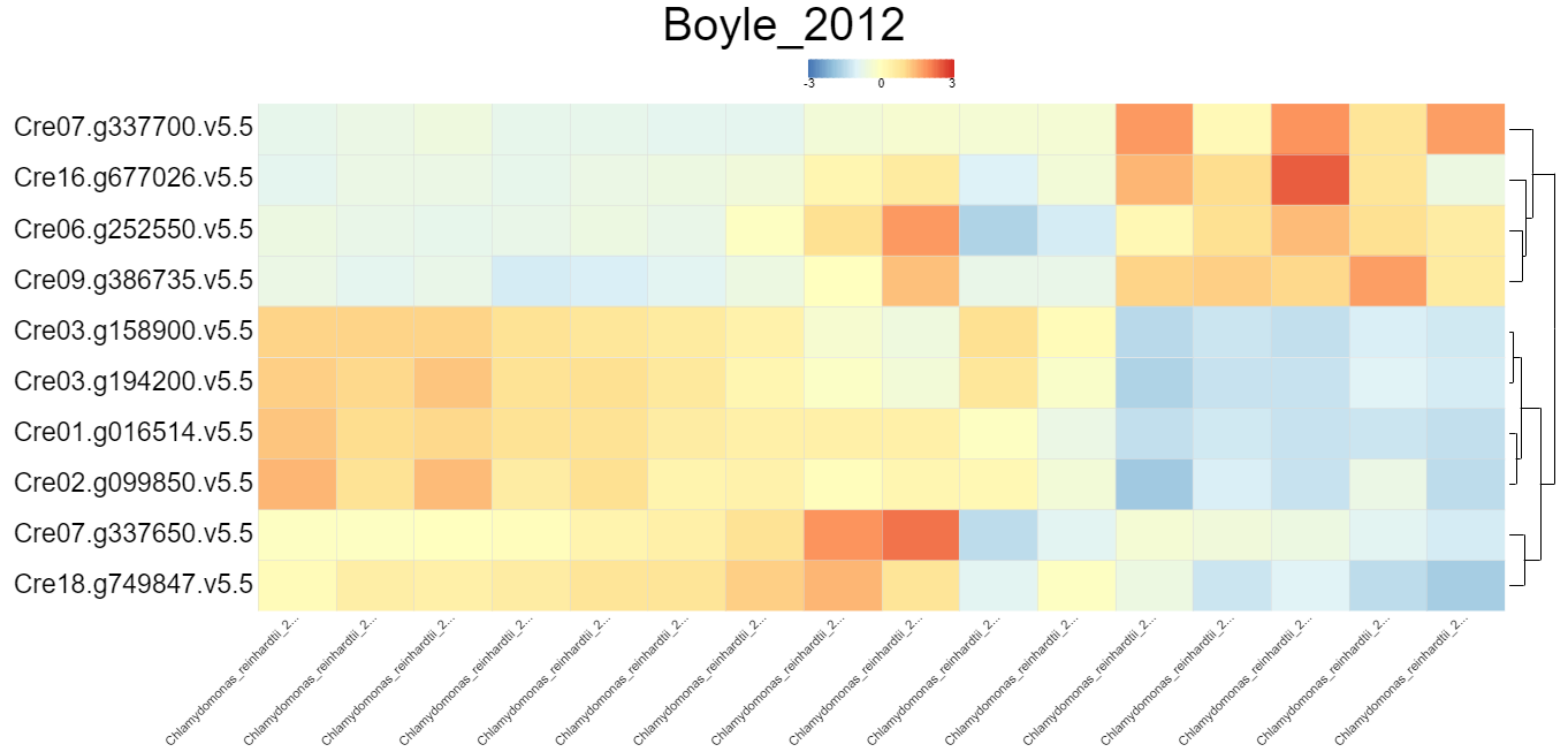

Supplement: Supplementary file 1 [file cells-12-01379-s001.zip › Figure S11, Boyle et al. (2012) -N transcriptomics dataset. Pathway = acetyl-CoA synthesis from pyruvate.png]

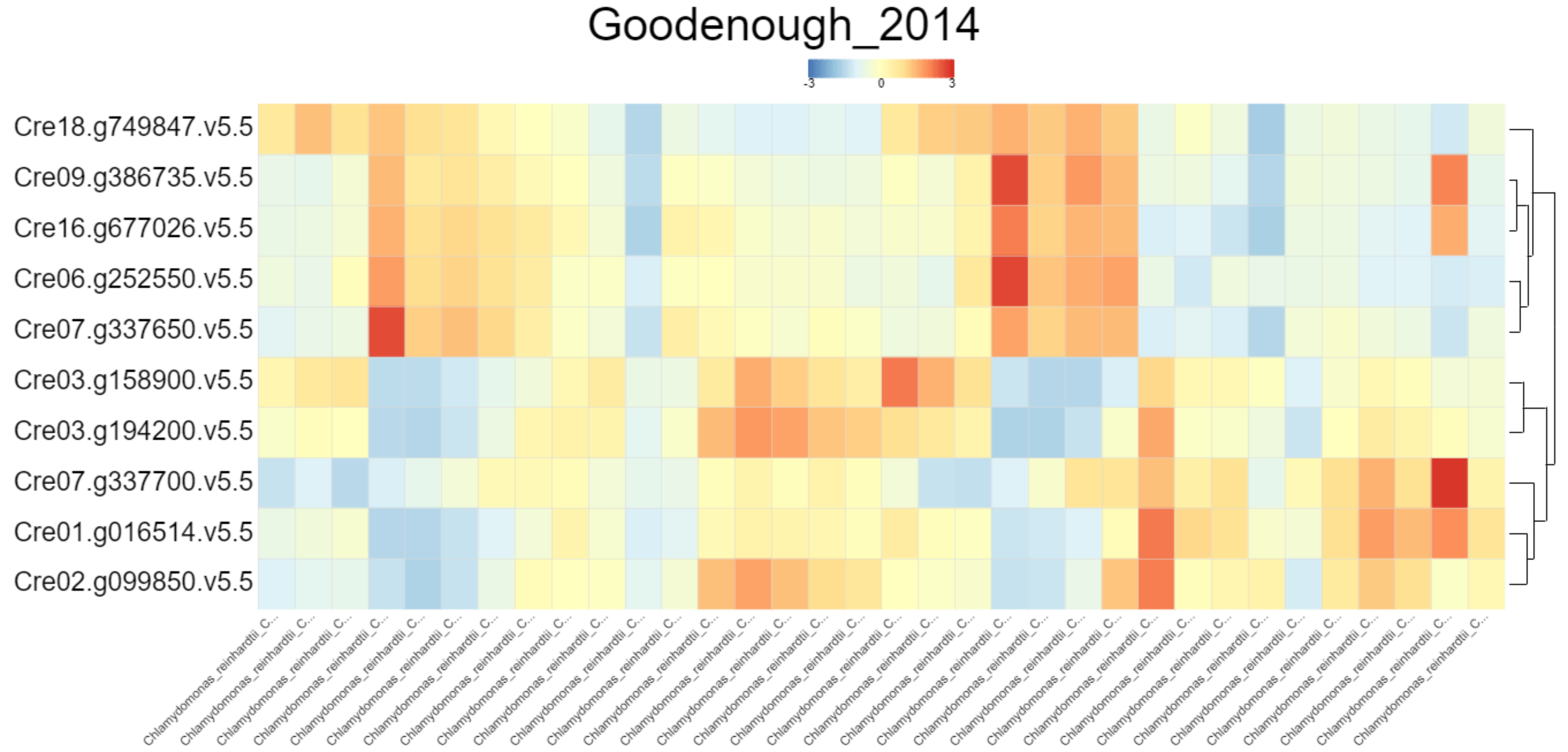

Supplement: Supplementary file 1 [file cells-12-01379-s001.zip › Figure S12, Goodenough et al. (2014) -N transcriptomics dataset. Pathway = acetyl-CoA synthesis from pyruvate.png]

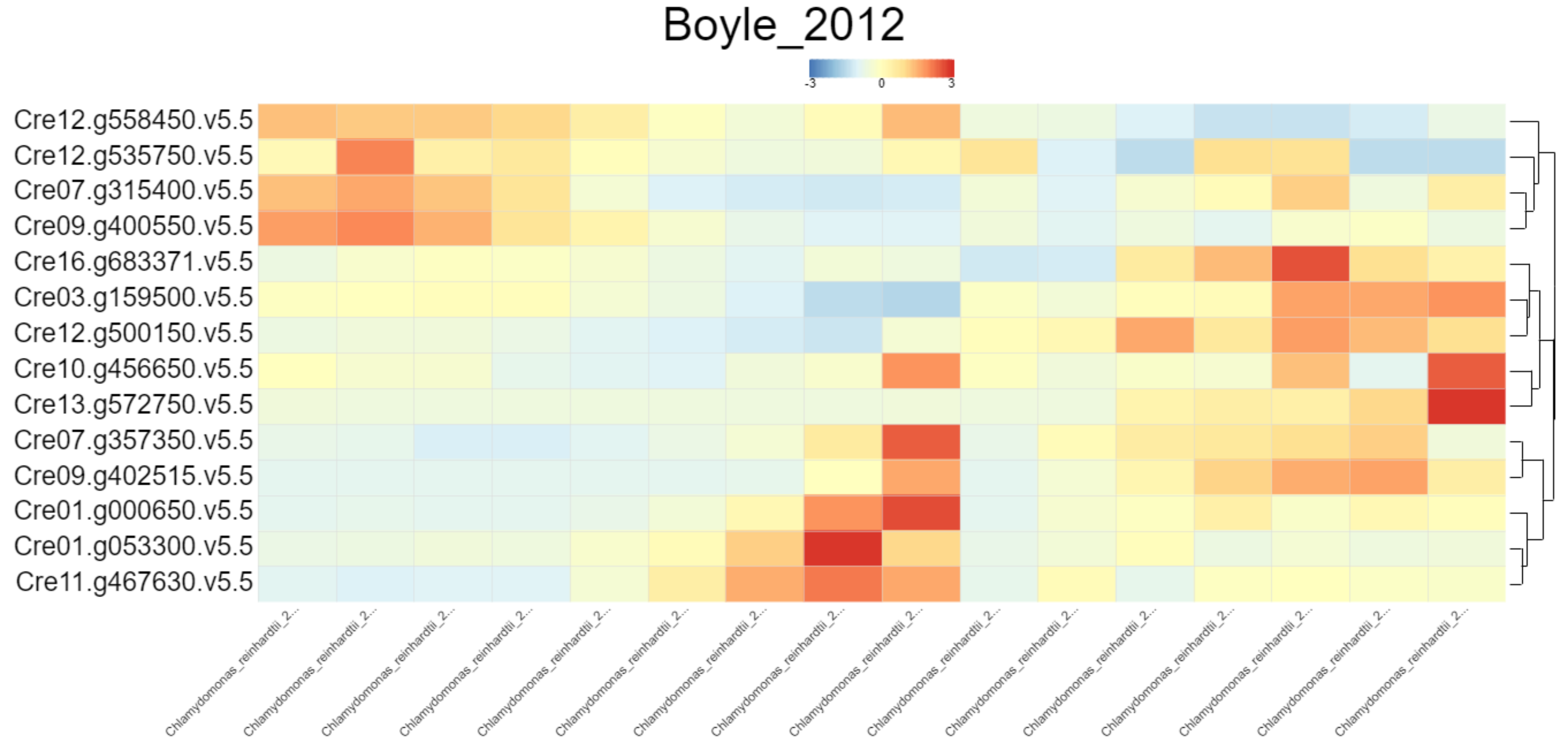

Supplement: Supplementary file 1 [file cells-12-01379-s001.zip › Figure S2, Boyle et al. (2012) -N transcriptomics dataset. Pathway = Arg catabolism to polyamines and NO.png]

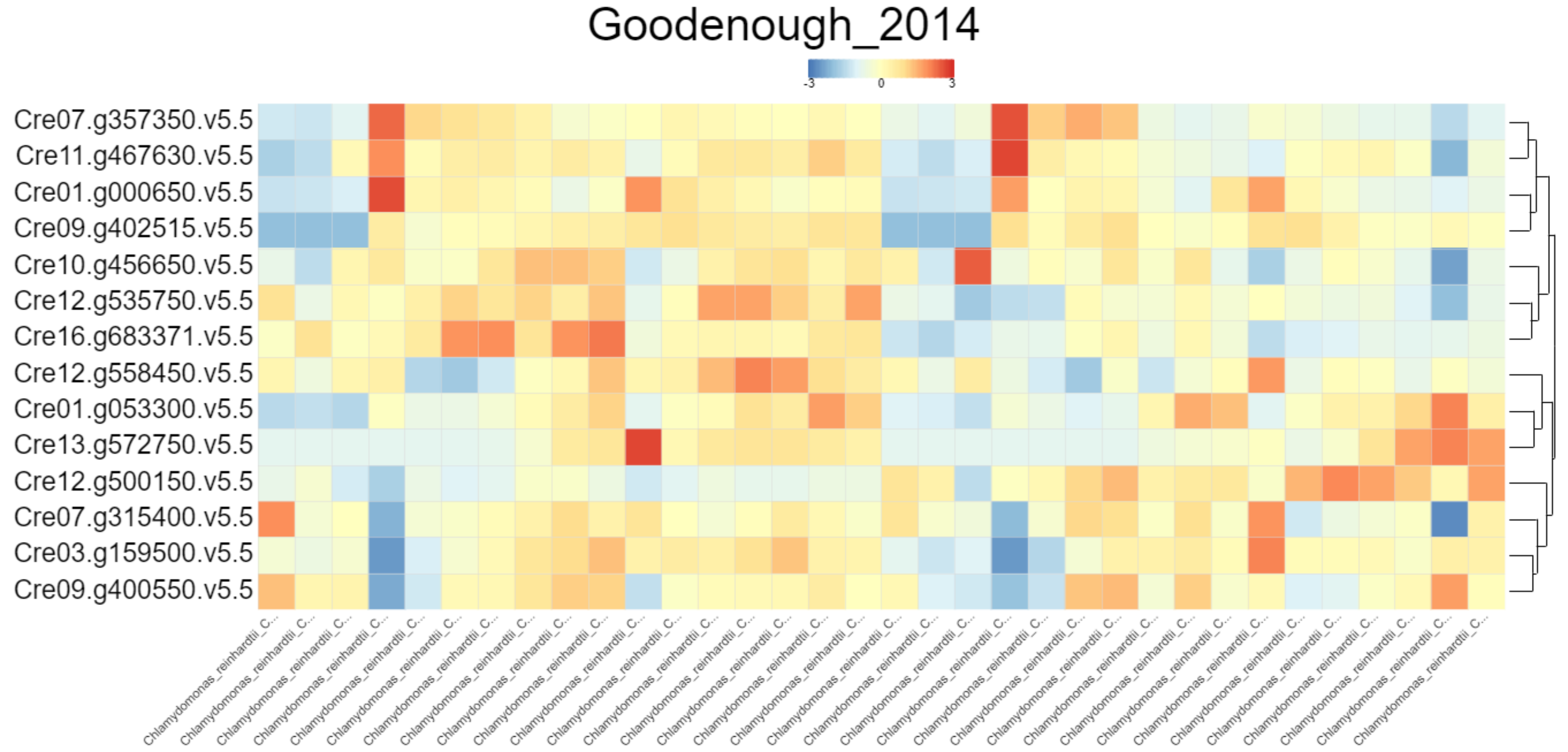

Supplement: Supplementary file 1 [file cells-12-01379-s001.zip › Figure S3, Goodenough et al. (2014) -N transcriptomics dataset. Pathway = Arg catabolism to polyamines and NO.png]

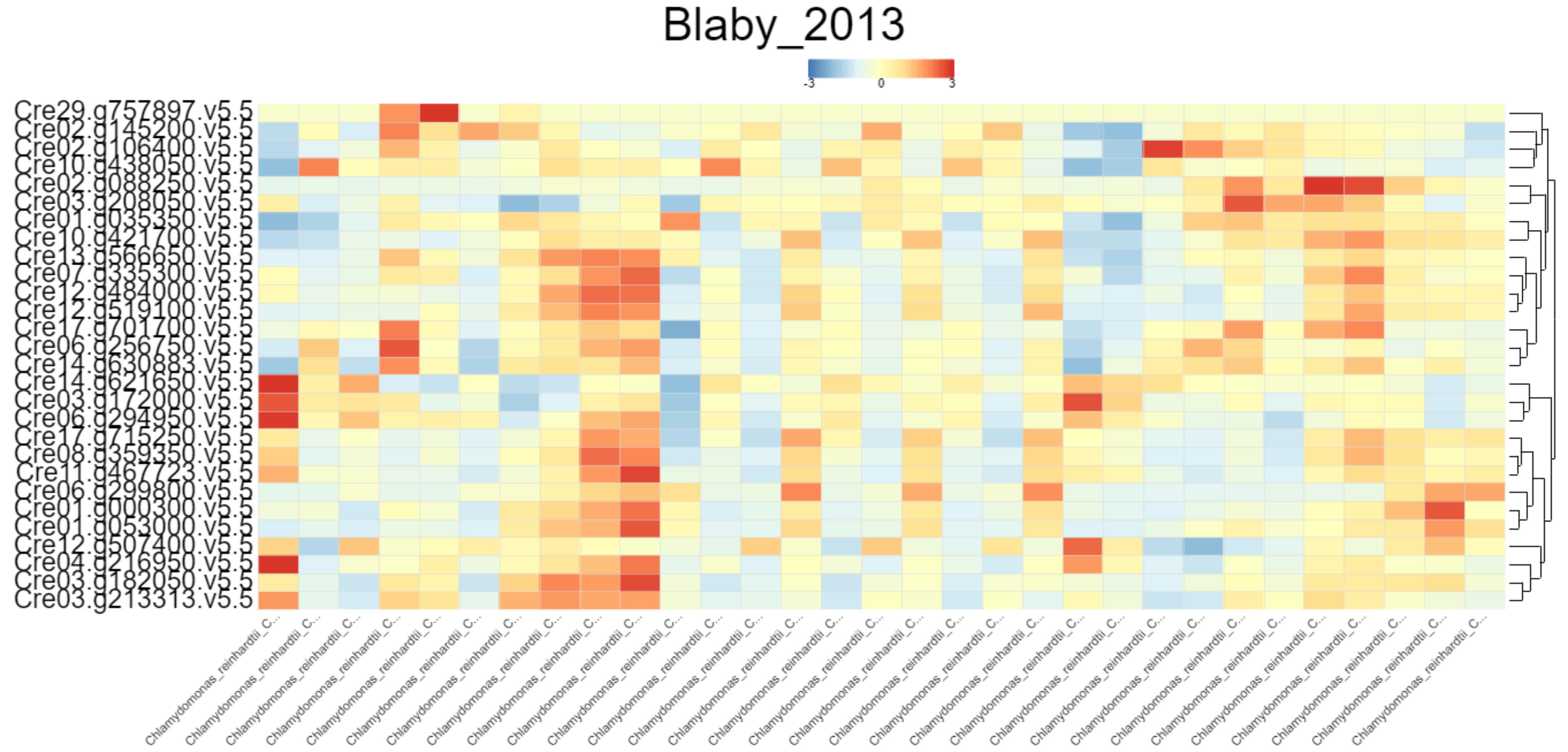

Supplement: Supplementary file 1 [file cells-12-01379-s001.zip › Figure S4, Blaby et al. (2013) -N transcriptomics dataset. Pathways = de novo fatty acid and TAG synthesis.png]

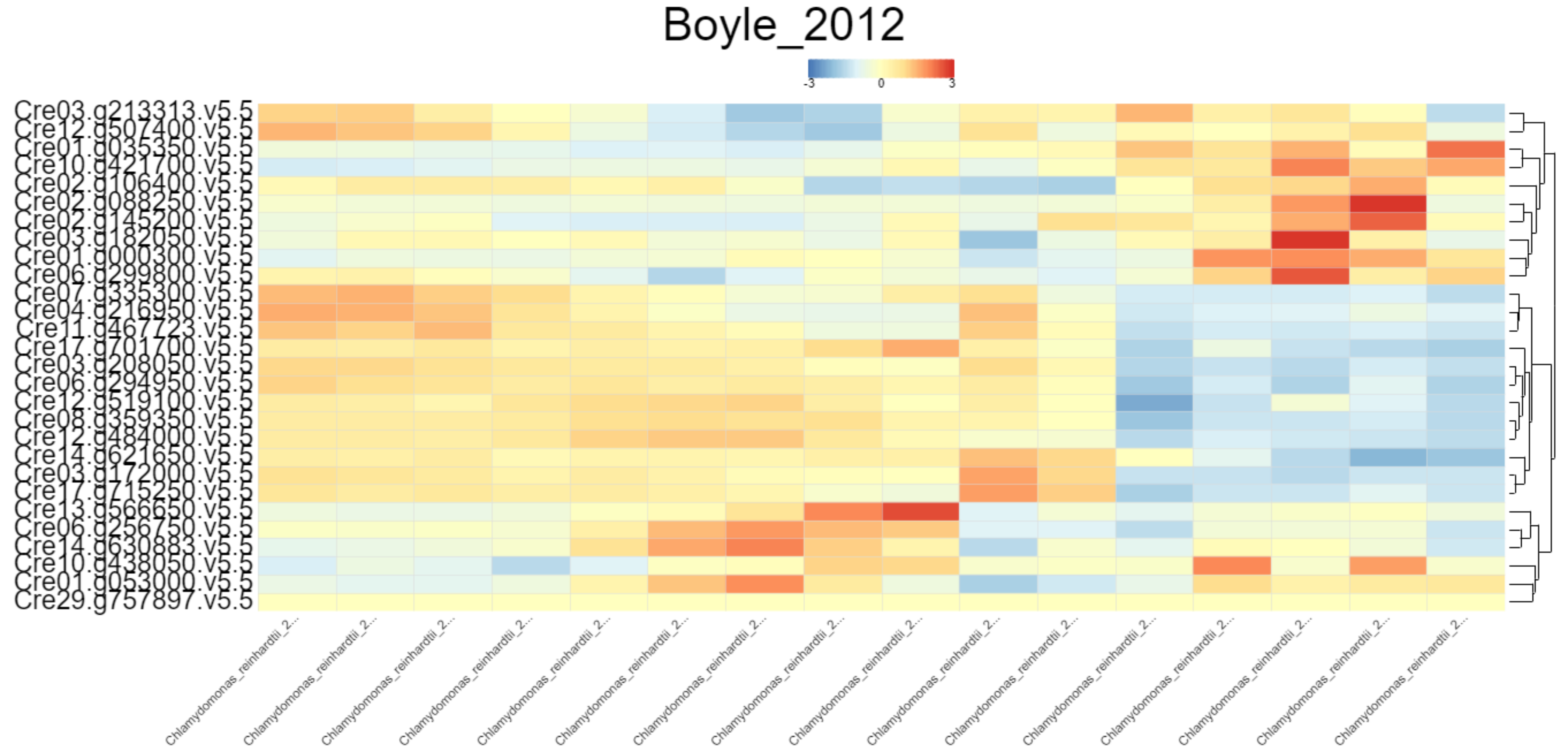

Supplement: Supplementary file 1 [file cells-12-01379-s001.zip › Figure S5, Boyle et al. (2012) -N transcriptomics dataset. Pathways = de novo fatty acid and TAG synthesis.png]

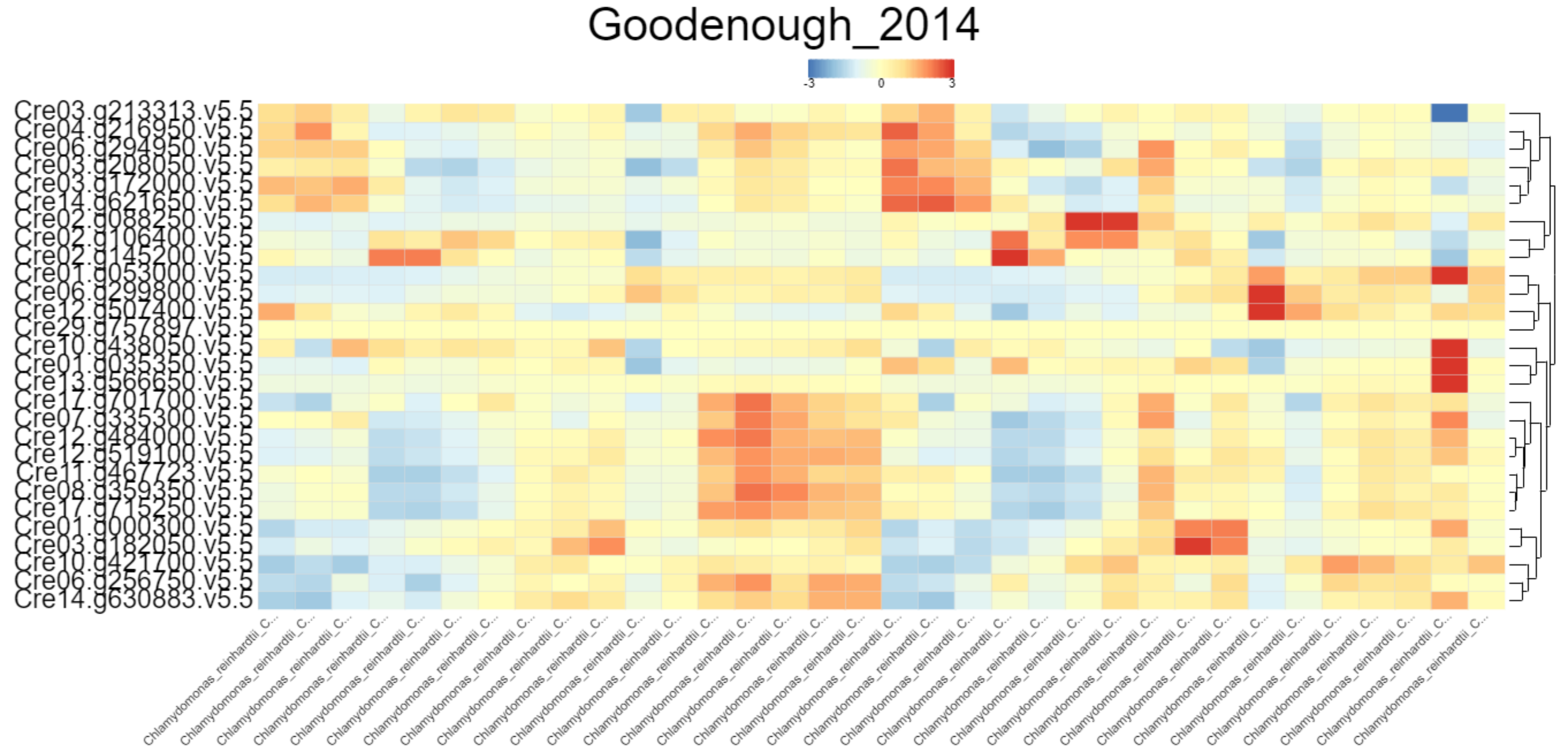

Supplement: Supplementary file 1 [file cells-12-01379-s001.zip › Figure S6, Goodenough et al. (2014) -N transcriptomics dataset. Pathways = de novo fatty acid and TAG synthesis.png]

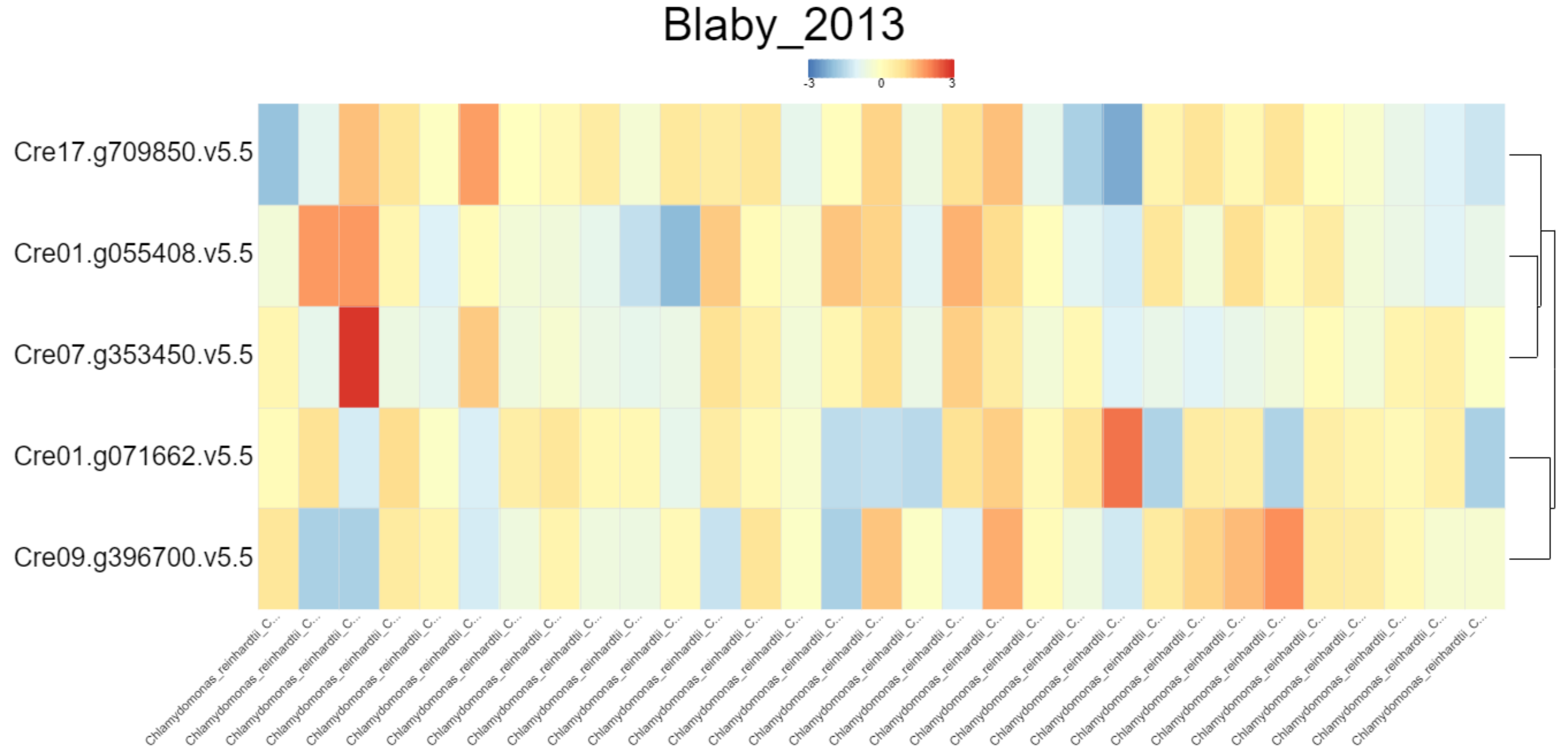

Supplement: Supplementary file 1 [file cells-12-01379-s001.zip › Figure S7, Blaby et al. (2013) -N transcriptomics dataset. Pathway = acetate assimilation.png]

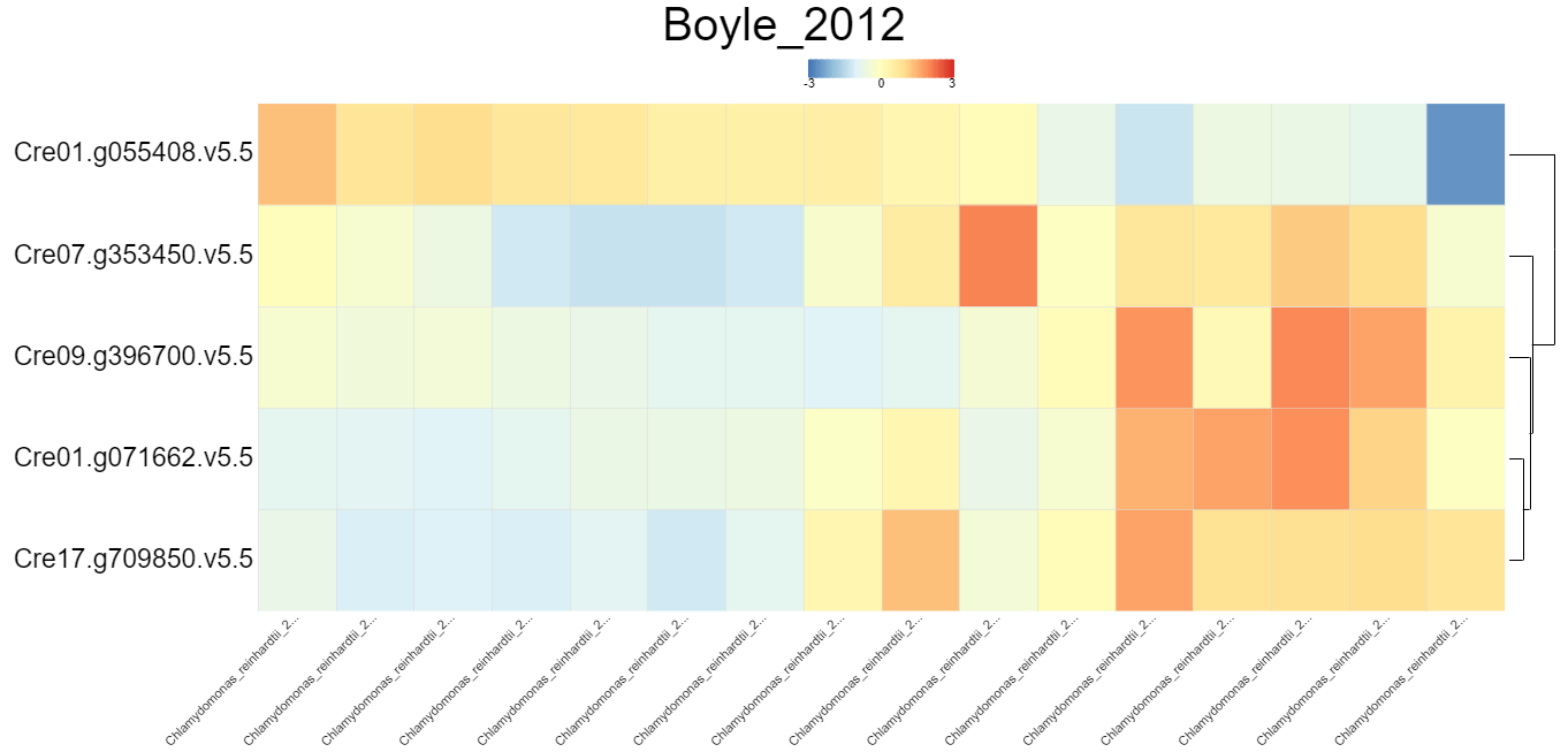

Supplement: Supplementary file 1 [file cells-12-01379-s001.zip › Figure S8, Boyle et al. (2012) -N transcriptomics dataset. Pathway = acetate assimilation.png]

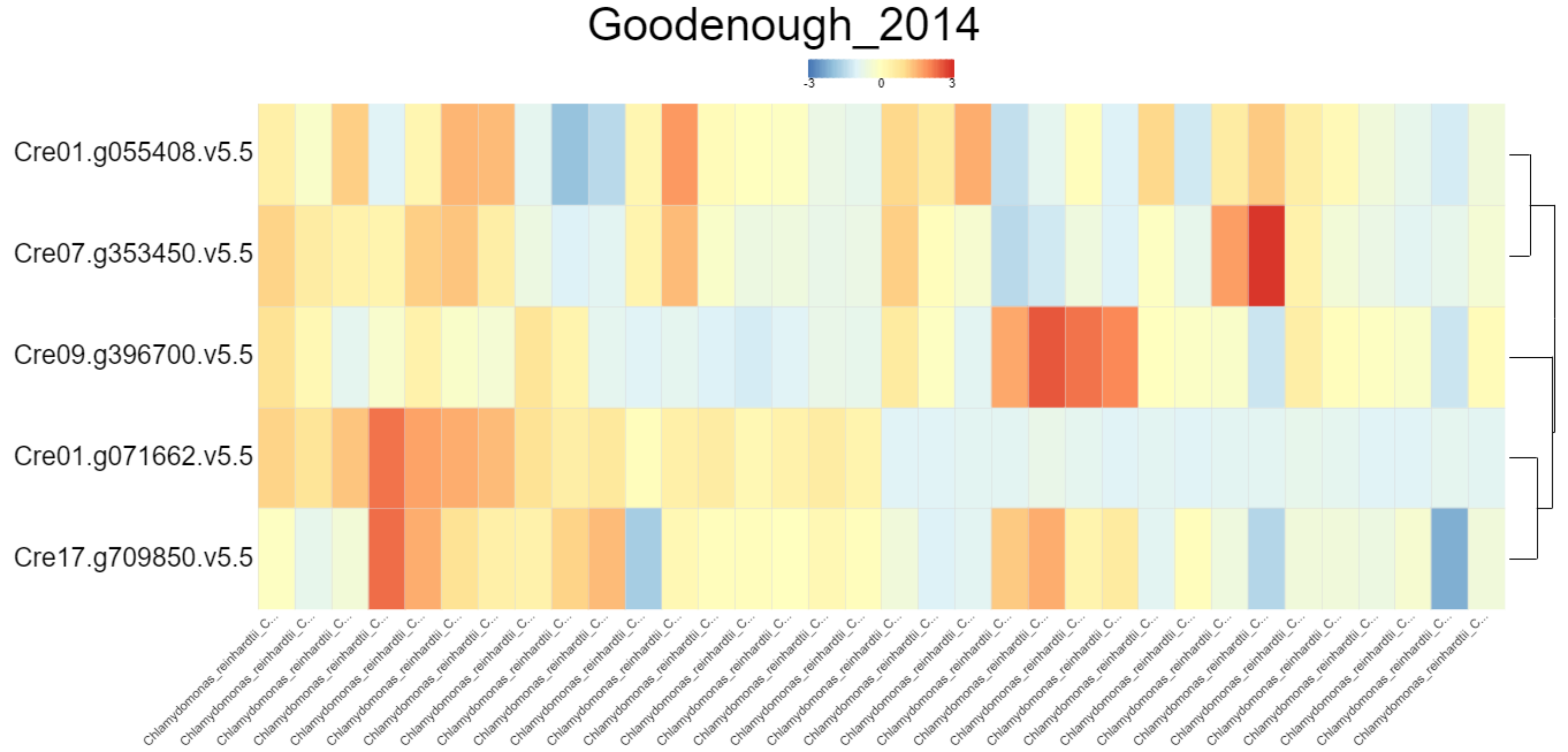

Supplement: Supplementary file 1 [file cells-12-01379-s001.zip › Figure S9, Goodenough et al. (2014) -N transcriptomics dataset. Pathway = acetate assimilation.png]
